# Association of *Helicobacter pylori* infection with the metabolic syndrome among HIV-infected black Africans receiving highly active antiretroviral therapy

**DOI:** 10.5830/CVJA-2015-012

**Published:** 2015

**Authors:** Benjamin Longo-Mbenza, Teke Apalata, Murielle Longokolo, Marcel Mbula Mambimbi, Mokondjimobe Etienne, Baudouin Buassa-bu-Tsumbu, Thierry Gombet, Bertrain Ellenga, Guy Milongo Dipa, Evelyne Lukoki Luila, Augustin Nge Okwe

**Affiliations:** Research Unit, Faculty of Health Sciences, Walter Sisulu University, Mthatha, South Africa; Department of Medical Microbiology, Faculty of Health Sciences, Walter Sisulu University, Mthatha, South Africa; Department of Internal Medicine, University of Kinshasa, Democratic Republic of the Congo; Department of Internal Medicine, University of Kinshasa, Democratic Republic of the Congo; Laboratoire de Biochimie et Pharmacologie, Faculté des Sciences de Santé, Brazzaville, Congo; Laboratoire de Biochimie et Pharmacologie, Faculté des Sciences de Santé, Brazzaville, Congo; Centre Hospitalier Universitaire, Faculté des Sciences de Santé, University of Marien Ngouabi, Brazzaville, Congo; Centre Hospitalier Universitaire, Faculté des Sciences de Santé, University of Marien Ngouabi, Brazzaville, Congo; Biostatistic Unit, Lomo Medical Cardiovascular Centre for Africa, Limete, Kinshasa, Democratic Republic of the Congo; Biostatistic Unit, Lomo Medical Cardiovascular Centre for Africa, Limete, Kinshasa, Democratic Republic of the Congo; Biostatistic Unit, Lomo Medical Cardiovascular Centre for Africa, Limete, Kinshasa, Democratic Republic of the Congo

**Keywords:** metabolic syndrome, Helicobacter pylori, HIV, HAART

## Abstract

**Introduction:**

The metabolic syndrome (MetS) is common in human immune deficiency virus (HIV)-infected individuals receiving highly active antiretroviral therapy (HAART). Immune deficiencies caused by HIV give rise to numerous opportunistic gastrointestinal pathogens such as *Helicobacter pylori*, the commonest cause of chronic gastritis. The study sought to determine the relationship between *H pylori* infection and the MetS among HIV-infected clinic attendees.

**Methods:**

This cross-sectional study was carried out in a specialised heart clinic in Kinshasa, DR Congo. Between January 2004 and December 2008, 116 HIV-infected patients (61 with MetS and 55 without MetS) who underwent upper gastrointestinal endoscopy for dyspeptic symptoms were included in the study following an informed consent. Univariate associations were determined by odds ratios (OR), while multivariate logistic regression analysis was used to identify factors associated with the MetS.

**Results:**

*H pylori* infection (OR = 13.5, 95% CI: 10.3–17.6; *p* < 0.0001) and peripheral obesity (median hip circumference ≥ 97 cm) (OR = 4.7, 95% CI: 1.2–18.8; *p* = 0.029) were identified as MetS-related factors in HIV-infected patients. Higher rates of the MetS were associated with increased incidence of HIV-related immunocompromise using World Health Organisation (WHO) staging criteria. There was a univariate significant difference in the prevalence of the MetS between antiretroviral therapy (ART)-naïve patients and patients treated by means of a first-line HAART regimen of stavudine (d4T), lamivudine (3TC) and nevirapine (NVP). However, this difference was not significant in multivariate logistic analysis.

**Conclusion:**

*H pylori* infection was significantly associated with the MetS in HIV-infected patients.

## Abstract

Developing countries have recently been experiencing an increase in the prevalence of risk factors associated with the metabolic syndrome (MetS) and cardiovascular diseases (CVD) in both general and working class populations.^1^-^5^ Progressive urbanisation and westernisation of lifestyle leading to an epidemiological transition in developing countries can be mentioned among possible reasons.^1^,^2^

The metabolic syndrome is a cluster of metabolic and haemodynamic risk factors that act multiplicatively to promote atherosclerotic CVD and type 2 diabetes mellitus (T2DM).^6^,^7^ Its initial description by Reaven (1988) included dyslipidaemia, hypertension, hyperglycaemia and insulin resistance.^8^ These disturbances promote an elevated prothrombotic/procoagulant state, endothelial cell dysfunction and inflammatory response with premature atherosclerotic complications.^9^,^10^

*Helicobacter pylori*, a spiral-shaped gram-negative flagellated bacterium, enhances human chronic inflammatory diseases.^11^ Epidemiological data from the literature support a significant association of *H pylori* seropositivity with CVD, insulin resistance and elevated parameters of the metabolic syndrome.^12^,^13^

The risk of the MetS is greater in HIV-infected individuals compared with the general population because of a greater prevalence of lipid and glucose abnormalities.^14^,^15^ HIV infection itself is associated with disturbances in lipid metabolism such as hyperglyceridaemia, and a decrease in total cholesterol and high-density lipoprotein (HDL) cholesterol levels.^16^ Treatment of HIV infection with highly active antiretroviral therapy (HAART) can also induce severe metabolic complications including lipodystrophy, dyslipidaemia, and insulin resistance. Patients with HIV infection and the MetS had increased intima–media thickness (IMT), similar to that found in diabetes.

While inflammation is recognised as a major contributor in the pathogenesis of both diabetes and atherosclerosis, little is known about the key inflammatory molecules involved in atheroma and diabetes in HIV-positive HAART recipients. However, epidemiological studies have shown that *H pylori* infection has become a common cause of chronic gastritis in HIV/AIDS patients.^17^ It is possible that prevalent infection by *H pylori* enhances the inflammatory process observed in the atheroma of HAART-recipient HIV-positive individuals, leading to CVD and the MetS.

In many central African countries, the first-line anti-retroviral therapy (ART) protocol in the public health sector recommends the combination of three drugs (stavudine, lamivudine and efavirenz), commonly referred to as ‘regimen 1A’. In ‘regimen 1B’, efavirenz is substituted with nevirapine, particularly in females of reproductive age.

There is a paucity of data on *H pylori* seropositivity, socio-economic status and the use of HAART in patients with the MetS and HIV co-infection among black Africans. Hence, the aim of this study was to determine the relationship between *H pylori* infection and the MetS among HIV-infected black Africans.

## Methods

This was a cross-sectional study design. The study population consisted of HIV-infected patients, aged 20 years and above; all black Africans attending LOMO specialised heart clinic in Kinshasa, Democratic Republic of the Congo between January 2004 and December 2008.

The study protocol was designed according to the Helsinki Declaration II,^18^ and approved by the local ethics committee. Patients were consecutively enrolled in the study if they were HIV infected, and diagnosed with or without the MetS.

Exclusion criteria included pregnancy, dysfunctional thyroid gland, nephrotic syndrome, hepatic cirrhosis, and use of any of beta-blocker, digoxine, lipid-lowering drugs or insulin. All study participants were enrolled by informed consent. Associations between *H pylori* infection and the MetS were assessed among HIV-infected patients with and without the MetS.

Data were collected using structured and standardised questionnaires. Demographic data (gender, age), lifestyle (socioeconomic status) and behavioural risk factors (intravenous drug use, current cigarette smoking and excessive alcohol intake) were recorded. Low and high socio-economic status (SES) were defined according to our previous work.^2^ Patients’ anthropometric parameters (body weight and height, waist and hip circumferences) were measured following a physical examination.

For patients diagnosed as having HIV infection, we used World Health Organisation (WHO)^19^ and Centres for Diseases Control and Prevention (CDC)^20^ staging systems to classify their disease stages. Information on the use of highly active anti-retroviral therapy (HAART) was obtained from all study participants.

Blood pressure (BP) was measured after the participant had rested for 10 minutes, seated in a quiet waiting room. BP was measured on the left arm with elbow flexed at heart level, by the same physician using an Omron HEM 705 electronic BP manometer (Omron Life Science Co, Ltd, Tokyo, Japan). Three readings were obtained, and the average was used for the analysis.

## Definitions and criteria for the MetS

Criteria defined by the 2005 International Diabetes Federations (IDF) report were used to ascertain cases of the MetS.^21^ Participants with three of the following criteria were defined as having the metabolic syndrome: prerequisite was waist circumference ≥ 94 cm in men and ≥ 80 cm in women; triglycerides ≥ 150 mg/dl (1.7 mmol/l); HDL cholesterol < 40mg/dl (1.03 mmol/l) in men and < 50 mg/dl (1.29 mmol/l) in women; systolic blood pressure ≥ 130 mmHg, diastolic blood pressure ≥ 85 mmHg; and fasting glucose ≥ 100 mg/dl (5.6 mmol/l) or previously diagnosed type 2 diabetes. Other participants met the criteria for high blood pressure or high fasting glucose levels if they were currently on antihypertensive or oral hypoglycaemic therapies, respectively.

The cardiometabolic co-morbidities included arterial hypertension, type 2 diabetes, myocardial infarction, stroke, long QTc ≥ 0.420 ms, gout/hyperuricaemia (uric acid ≥ 7 mg/dl), and subclinical atherosclerosis (pulse pressure ≥ 60 mmHg + IMT ≥ 1 mm or carotid plaque).^4^,^22^,^23^

## Laboratory investigations

The initial HIV test was performed using HIV rapid test (SmartCheck test, World Diagnostics Inc, USA) while a confirmatory test following an initial positive HIV result was performed using Uni-Gold^TM^ Recombigen® HIV (Trinity Biotech PLC, USA) from the blood samples. CD4^+^ lymphocyte cell count was measured using CyFlowR Counter (Partec GmbH; Munstar, Germany) and HIV RNA viral load was quantified by means of Nuclisens Easy Q HIV-1 system (Biomérieux, Box tel, the Netherlands).

Haemoglobin and haematocrit levels were measured in blood using standard haematological techniques. Fasting glucose levels were measured from plasma samples using the glucoseoxydase method and spectrophotometer (Hospitex Diagnostics, Florence, Italy). Total cholesterol, HDL cholesterol, uric acid and triglyceride levels were measured using enzymatic colorimetric methods (Biomérieux, Marcy l’Etoile, France). Oxidised low-density lipoprotein (LDL) cholesterol, a biomarker of oxidative stress, was measured using solid-phase two-side enzyme immunoassay (Mercodia AB, Sylveniusgatan 8A, SE754 50, Uppsala, Sweden).

*H pylori* infection was assayed by the determination of immunoglobulin G (IgG) antibodies as described elsewhere.^4^ Briefly, IgG antibodies to *H pylori* (anti-HP Ab) were measured by a commercial enzyme-linked immunosorbent assay (Pyloriset® EIA-G; Orion Diagnostica, Espoo, Finland). The detection range of serum levels of anti-HP Ab assay was between 100 and 12 800 U.

## Imaging techniques

Subclinical atherosclerosis was assayed by IMT using echo-Doppler, and the diagnosis of *H pylori*-related chronic gastritis was confirmed as described elsewhere.4 Atherosclerotic complications including different forms of CVD (myocardial infarction, stroke, peripheral artery disease) were ascertained by clinical symptoms and signs, cardiac enzymes and tropinin levels, as well as results from electrocardiogram, echo-Doppler, tomodensitometry and coronary angiogram.

## Statistical analyses

Data were expressed as means ± standard deviation (SD) for the continuous variables and proportions (percentages) for the categorical variables. The Student’s *t*-test was performed to assess differences between two means and ANOVA between groups. When data were not normally distributed, the Mann–Whitney *U*-test was used. Either the chi-square test with and without trend or Fischer’s exact test was used to test the degree of association of categorical variables.

Variables were first computed to identify univariate potential factors and cardiometabolic co-morbidities associated with the MetS; the significant association between variables being calculated as odds ratios (OR) with 95% confidence interval (CI). Potential factors demonstrating a univariate relationship (*p* < 0.20) with the MetS were included in the multivariate logistic regression analysis to assess the effect of their independent association with the MetS. Goodness-of-fit was verified with the Hosmer and Lemeshow statistical method. A *p*-value < 0.05 was considered statistically significant. All data were analysed using the Statistical Package for the Social Sciences (SPSS for Windows, version 21; Chicago, IL).

## Results

A total of 116 heterosexual HIV-infected patients were enrolled. Of the 116 eligible study participants, 54 (46.6%) were men and 62 (53.4%) women. The mean age of the study participants was 42 ± 9 years. Of these, 65 (56%) were ART naïve and 51 (44%) were on a 13 ± 1 month first-line HAART regimen of stavudine (d4T), lamivudine (3TC) and nevirapine (NVP). No patient received either efavirenz or protease inhibitors. Based on the 2005 International Diabetes Federation definition, 61/116 patients (52.6%) met the criteria for the MetS versus 55/116 patients (47.4%) without the MetS.

During univariate analyses, numerous factors were shown to be significantly associated with the MetS in HIV-infected individuals, as depicted in Tables 1 and 2. There was a significant univariate association in the prevalence of the MetS between ART-naïve patients and those treated by means of a first-line HAART regimen of d4T, 3TC and NVP (Table 1) but this association did not reach significant difference in multivariate regression analysis.

**Table 1:** Univariate factors associated with the metabolic syndrome in HIV-infected individuals (*n* = 116)

*Variable of interest*	*Presence of MetS n (%)*	*Absence of MetS n (%)*	*OR (95% CI)*	*p-value*
Gender			1.4 (0.7 – 3)	0.332
males (*n* = 54)	31 (57.4)	23 (42.6)		
females (*n* = 62)	30 (48.4)	32 (51.6)		
Socio-economic status (SES)			3.3 (1.4 – 7.8)	0.004
high (*n* = 36)	26 (72.2)	10 (27.8)		
low (*n* = 80)	35 (43.8)	45 (56.2)		
Smoking			10.5 (2.9 – 37.9)	<0.0001
yes (*n* = 26)	23 (88.5)	3 (11.5)		
no (*n* = 90)	38 (42.2)	52 (57.8)		
*Helicobacter pylori seropositivity*			95.3 (20.4 – 444.7)	<0.0001
yes (*n* = 72)	59 (81.9)	13 (18.1)		
no (*n* = 44)	2 (4.5)	42 (95.5)		
chronic gastritis due to *H pylori*			28.1 (9.5 – 83)	<0.0001
yes (*n* = 50)	45 (90)	5 (10)		
no (*n* = 66)	16 (24.2)	50 (75.8)		
Peripheral obesity (median hip circumference ≥ 97 cm)			4.6 (2.1 – 10)	<0.0001
yes (*n* = 58)	41 (70.7)	17 (29.3)		
no (*n* = 58)	20 (32.8)	38 (67.2)		
Excessive alcohol intake			3.3 (1.5 – 7.4)	0.003
yes (*n* = 44)	31 (70.5)	13 (29.5)		
no (*n* = 72)	30 (41.7)	42 (58.3)		
HAART exposure			2.4 (1.01 – 5.7)	0.045
yes (*n* = 65)	34 (52.3)	31 (47.7)		
no (*n* = 35)	11 (31.4)	24 (68.6)		

HAART = highly active antiretroviral therapy; MetS = metabolic syndrome; OR = odds ratio; CI = confidence interval.

**Table 2:** Other univariate factors associated with metabolic syndrome in HIV-infected individuals (*n* = 116)

*Variables of interest*	*Presence of Mets Mean ± SD*	*Absence of Mets Mean ± SD*	*p-value ANOVA*
Age (years)	46.4 ± 8	40.8 ± 11.1	0.005
BMI (kg/m²)	23.1 ± 4.4	20.5 ± 4.1	0.003
WC (cm)	109.2 ± 16.8	90 ± 16.6	< 0.0001
HC (cm)	111.6 ± 13.7	103.2 ± 16.3	0.013
SBP (mmHg)	138.7 ± 25.1	114.5 ± 21	< 0.0001
DBP (mmHg)	77.1 ± 12.9	72.3 ± 12.2	0.068
Pulse pressure (mmHg)	61.6 ± 23.2	42.2 ± 13.2	< 0.0001
Haemoglobin (g/dl)	13.7 ± 1.1	12.2 ± 1.8	0.005
Haematocrit (%)	36.8 ± 5.9	28.2 ± 7.4	< 0.0001
IgG H pylori (U/ml)	394.6 ± 61.1	126.9 ± 192.1	< 0.0001
CD4+ count (cells/mm3)	199.5 ± 157.9	181.5 ± 193.9	0.026**
Viral load (copies/ml)	270373 ± 147064	208741 ± 102629	< 0.0001**
Uric acid (mg/dl)	33.9 ± 10.2	10.7 ± 10.8	< 0.0001
Fasting glucose (mg/dl)	130.1 ± 26.4	106.4 ± 50.2	0.008
(mmol/l)	7.22 ± 1.47	5.91 ± 2.79	
Total cholesterol (mg/dl)	193.9 ± 51.9	157.6 ± 79.8	0.018
(mmol/l)	5.02 ± 1.34	4.08 ± 2.07	
HDL-C (mg/dl)	78.5 ± 26.6	70.4 ± 16.8	0.084
(mmol/l)	2.03 ± 0.69	1.82 ± 0.44	
Triglycerides (mg/dl)	255.8 ± 41.7	206.8 ± 69.5	0.009
(mmol/l)	2.89 ± 0.47	2.34 ± 0.79	
Oxidised LDL-C (mg/dl)	155.1 ± 0.3	101.2 ± 0.1	< 0.0001
(mmol/l)	4.02 ± 0.01	2.62 ± 0.00	

**Non-parametric Mann–Whitney *U*-test

**Fig. 1.**
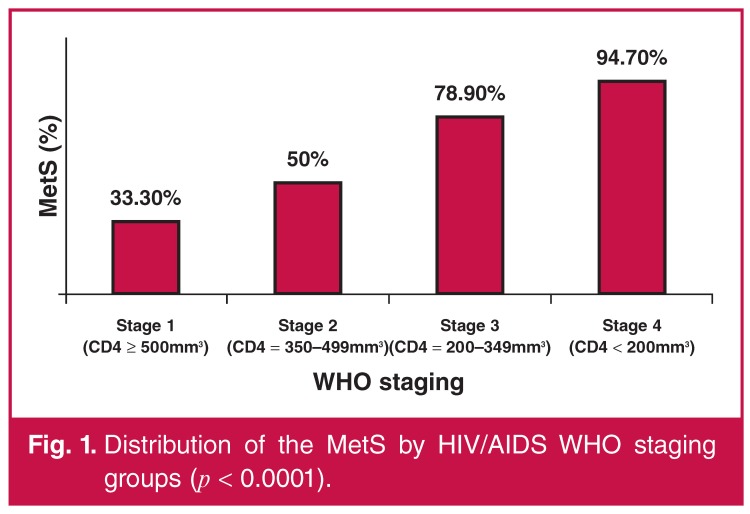
Distribution of the MetS by HIV/AIDS WHO staging groups (*p* < 0.0001).

When using non-parametric Mann–Whitney *U*-tests, there were significant univariate associations of CD4^+^ T cell counts and HIV viral loads with the MetS (Table 2). There was also a significant relationship (*p* < 0.0001) between the WHO HIV disease stages and the presence of the MetS (Fig. 1). HIV-infected patients of WHO stages 3 and 4 were in CDC stage C and those of WHO stages 1 and 2 were in CDC stage B.

However, during multivariate logistic regression analysis, after adjusting for age, SES, HAART exposure, smoking, excessive alcohol intake, waist circumference, CD4^+^ T-cell counts and plasma HIV loads, *H pylori* seropositivity (constant B = 5.2; SE = 1.114; wald χ^2^ = 21.785; OR = 13.5, 95% CI: 10.3–17.6; *p* < 0.0001) and peripheral obesity (median hip circumference ≥ 97 cm) (constant B = 1.545; SE = 0.708; wald χ^2^ = 4.756; OR = 4.7, 95% CI: 1.2–18.8; *p* = 0.029) were identified as the only factors significantly associated with the MetS in HIV-infected patients.

## Discussion

The metabolic syndrome is recognised as a major public health concern, even in the absence of HIV infection.^4^,^6^,^21^,^24^ The majority of patients with the MetS were defined by high SES, physical inactivity, excessive alcohol intake, and total and peripheral obesity.^6^,^25^ In Africa, many individuals gain weight later in their adult life and do not want to loose weight because of the stigma of HIV.^24^ Furthermore, abdominal obesity is considered a social achievement.

Lifestyle has a strong influence on the MetS, particularly among HIV-infected patients. Therefore the main emphasis in the management of the MetS should focus on addressing lifestyle changes, mainly efforts to stop smoking, reduce body weight and alcohol intake, and increase moderate physical activity. Elevated blood pressure, dyslipidaemia and hyperglycaemia may however require additional drug treatment.

Additional correlates of the MetS among HIV-infected Africans in our study population were hypercoagulability, increased levels of uric acid, and infection/inflammatory markers, as reported in other study cohorts of both HIV-infected and uninfected patients.^4^,^5^,^26^ *Helicobacter pylori* infection and hip circumference ≥ 97cm (peripheral obesity) were identified as the only factors associated with the MetS in our study population during a multivariate analysis.

Findings from this study showed only univariate association between exposure to first-line combination antiretroviral therapy and the MetS. A previous report from the literature has underlined the independent role of stavudine (d4T as a part of ARV) in determining the MetS in HIV-infected populations.^26^ A possible contribution of the nucleoside analogue stavudine to lipid abnormalities was also previously reported in the literature.^27^ Numerous other studies confirmed that non-nucleoside reverse transcriptase inhibitors had a more favourable impact on lipid levels than most members of the protease inhibitor class.^24^,^28^,^29^

In addition, higher frequency of coronary heart disease,^30^ stroke^31^ and diabetes mellitus^32^ have been observed by others in HIV/AIDS patients with the MetS. Oxidative stress-mediated LDL cholesterol modification may be a key role player in initiation and exacerbation of the MetS and atherosclerosis in these HIV-infected patients.

Findings from this present study have supported the association between *H pylori* infection and larger hip circumference (≥ 97 cm). Appropriate lifestyle changes and in some cases, medication (antibiotics, statins, antihypertensives, antidiabetic drugs) may improve all of the MetS components. Getting more physical activity, losing weight (5–10% of weight), quitting smoking, limiting alcohol intake and appropriate diet (vitamins, antioxidants, fruits, vegetables, fish and whole grains) could be proposed to patients with the MetS.

Limitations of this study are mainly the small size of the study sample, the cross-sectional study design, and absence of an HIV-negative group. In this regard, results reported herein are only associations from which no conclusions regarding causality can be drawn.

## Conclusion

*H pylori* infection and peripheral obesity (median hip circumference ≥ 97 cm) were shown to be associated with higher risk of the MetS in HIV/AIDS patients. Screening for the presence of *H pylori* infection can be helpful when managing HIV/AIDS patients diagnosed with the MetS. However, further studies are warranted in order to ascertain the value of this recommendation.
